# Uses of mathematical modeling to estimate the impact of mass drug administration of antibiotics on antimicrobial resistance within and between communities

**DOI:** 10.1186/s40249-022-00997-7

**Published:** 2022-06-30

**Authors:** Scott W. Olesen

**Affiliations:** Department of Immunology and Infectious Diseases, Harvard Chan School, Boston, MA USA

**Keywords:** Mass drug administration, Azithromycin, Antibiotic resistance, Mathematical model

## Abstract

**Background:**

Antibiotics are a key part of modern healthcare, but their use has downsides, including selecting for antibiotic resistance, both in the individuals treated with antibiotics and in the community at large. When evaluating the benefits and costs of mass administration of azithromycin to reduce childhood mortality, effects of antibiotic use on antibiotic resistance are important but difficult to measure, especially when evaluating resistance that “spills over” from antibiotic-treated individuals to other members of their community. The aim of this scoping review was to identify how the existing literature on antibiotic resistance modeling could be better leveraged to understand the effect of mass drug administration (MDA) on antibiotic resistance.

**Main text:**

Mathematical models of antibiotic use and resistance may be useful for estimating the expected effects of different MDA implementations on different populations, as well as aiding interpretation of existing data and guiding future experimental design. Here, strengths and limitations of models of antibiotic resistance are reviewed, and possible applications of those models in the context of mass drug administration with azithromycin are discussed.

**Conclusions:**

Statistical models of antibiotic use and resistance may provide robust and relevant estimates of the possible effects of MDA on resistance. Mechanistic models of resistance, while able to more precisely estimate the effects of different implementations of MDA on resistance, may require more data from MDA trials to be accurately parameterized.

**Graphical Abstract:**

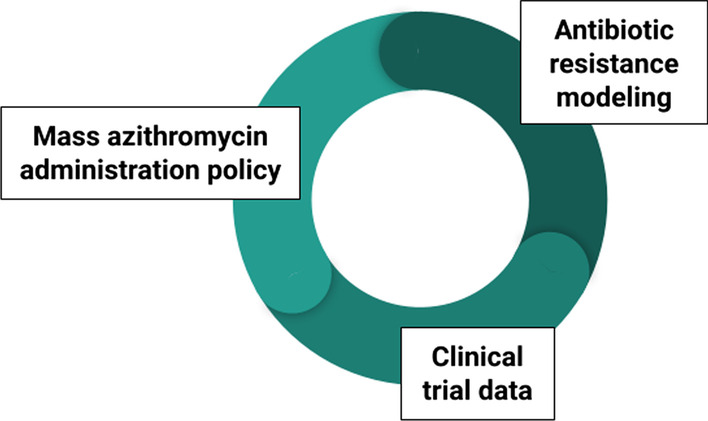

## Background

Mass drug administration (MDA) is the blanket treatment with anti-infectives of most people, or a select age group, in a target population like a settlement or an administrative region. MDA has been used for decades for control of parasites such as helminths and for control of bacteria such as *Chlamydia trachomatis* [1]. In 2020, based on results from a few key interventional trials [2–4], the World Health Organization made a recommendation that “consideration be given” to using azithromycin MDA to prevent child mortality, without targeting a specific pathogen, but only in a narrow context. First, MDA should only be used in sub-Saharan African settings with certain minimum infant mortality rates. Second, mortality rates, adverse effects of MDA, and antibiotic resistance must be continuously monitored as MDA is used. Finally, other child survival interventions must be in place in addition to MDA. In these circumstances, the recommended treatment is 2 azithromycin doses per year only for children aged 1–11 months [5].

Like any medical treatment, MDA has costs and benefits. Factors like the monetary cost of treatment and the risk of side effects must be weighed against the treatment’s therapeutic benefit [6, 7]. In the case of infectious diseases, costs and benefits must be weighed at the level of populations as well as at the level of individuals. For example, treating a disease in an individual may create a population-level benefit by preventing onward disease transmission or by even eliminating a disease altogether [8]. On the other hand, antibiotic therapy like MDA-azithromycin can promote antibiotic resistance among bacteria in treated individuals, which in turn could “spill over” into untreated individuals [9, 10]. Thus, a comprehensive cost-benefit analysis of MDA-azithromycin requires quantifying the degree to which MDA will promote antibiotic resistance in the treated individuals, within their community, and across other communities.

Ideally, clinical trials could fully characterize the effect of MDA-azithromycin on individual- and population-level antibiotic resistance. In practice, because of the complexity of bacterial transmission dynamics and the finite resources that can be devoted to clinical trials, there will be policy-relevant questions about the effect of MDA on resistance that cannot be directly addressed by empirical data [11, 12]. Fortunately, mathematical models can help bridge the gap between available empirical data and operational policy questions [13–15]. For example, empirical studies had measured the rate at which individuals re-acquired *C. trachomatis* after MDA had presumably cleared the pathogen from them. Mathematical modeling was then used to infer the minimum frequency of MDA to eliminate *C. trachomatis* carriage across a wide population [16, 17]. Although the relationship between antibiotic use, such as MDA-azithromycin, and antibiotic resistance is likely more complex than the relationship between drug use and disease elimination, models of antibiotic resistance can be similarly employed to link available empirical data with policy questions.

## Methods

The goal of this scoping review was to identify how the existing literature on antibiotic resistance modeling could be better leveraged to understand the effect of MDA on antibiotic resistance. Data for this review were initially identified through two searches of PubMed. The first search used the terms “antibiotic resistance” and “model[ing]”. The second search used “mass drug administration” and “antibiotic.” This search provided an initial set of studies relevant to the topic. References from the initially identified studies were also reviewed and included in this review if relevant. Only articles published in English were included. This review did not limit the included articles based on year of publication.

## Results

### Predicting population-level antibiotic resistance from antibiotic use

Hundreds of empirical studies have measured the association between antibiotic use and resistance. In a 2014 meta-analysis [18], 67% of 243 studies antibiotic use and resistance showed a positive association between use and resistance. 73% of studies analyzed the association at the level of the individual (rather than the region or country), 75% were conducted in Europe or the US, and the vast majority studied *Streptococcus*, *Staphylococcus*, or enteric bacteria like *Escherichia coli*. However, despite this substantial body of research, we still lack a definitive understanding of the relationship between an individual’s antibiotic use and the rates of antibiotic resistance in the wider population [14]. This gap is due at least in part to the complex epidemiology of population-level antibiotic use and resistance.

First, population-level resistance is not just the aggregate of individual-level resistance selected for by those individuals’ use of antibiotics. Instead, there is a complex interplay between individual-level antibiotic use and the transmission of susceptible and resistant bacteria [10, 19]. Resistance can “spill over” from treated individuals to their family members [20], and there is evidence for quantifiable spillover at larger scales [9, 10, 14]. For example, spillover may be crucial to patterns of β-lactam resistance in *S. pneumoniae*, the pathogen and antibiotic class most studied in population-level studies of antibiotic use and resistance [18, 21]. The treatment of children with acute otitis media using penicillins has been observed to select for β-lactam resistance among *S. pneumoniae* that cause pneumonia in older adults [22]. However, even in this well-studied case, the relationship between antibiotic use in one population and rates of resistance in another are poorly quantified. Although spillover plays some role following MDA, the quantitative magnitude of this effect is poorly understood and likely varies by geographic scale [9], by pathogen, and by antibiotic class [18, 23]. Better quantification of spillover could be critical to understanding the effects of MDA, as interactions between the MDA-treated population and a control population could lead MDA clinical trials to underestimate the effect of MDA on resistance [9].

Second, antibiotic resistance is not itself a pathogen; it is a feature of some members of a bacterial species. In some cases, when an antibiotic-resistant pathogen has minimal competition from the antibiotic-susceptible strains of the same species, conceptualizing “resistance” as a standalone pathogen is effective. For example, Donker et al. [24] evaluated the relevance of different geographical scales for the spread of carbapenem-resistant Enterobacteriaceae in the United Kingdom without explicitly accounting for any carbapenem-susceptible strains. More generally, however, competition between resistant and susceptible strains of the same bacterial species is likely critical to successful modeling of the association between antibiotic use and resistance [14].

Competition can occur within the human host, and recent models of resistance have demonstrated how this within-host competition can help explain a key feature of antibiotic resistance epidemiology, namely, the durable co-existence of antibiotic-resistance and -susceptible strains of the same bacterial species [25]. Competition also occurs between hosts: susceptibility and resistance both spill over between populations, with important implications for MDA [26]. For example, rates of macrolide resistance among *S. pneumoniae* and *E. coli* carried by recipients of MDA-azithromycin increase substantially after treatment [1, 27, 28] but then appear to wane in the succeeding months [29, 30]. Although this waning could be partly due to intra-individual effects, population-level effects likely play an important role: susceptible strains in untreated individuals can be transmitted to antibiotic-treated individuals [19, 31].

Third, use of one antibiotic can select for resistance to other antibiotics, because the same resistance mechanism provides resistance to those other antibiotics (i.e., cross-resistance) or because one genetic element can include multiple genes that provide resistance against multiple antibiotics (i.e., co-resistance) [27, 32]. More broadly, the use on one antibiotic can select for resistance to another antibiotic simply because a single bacterial strain is resistant to both antibiotics, even if the two resistance mechanisms are not genetically linked (i.e., co-selection) [33, 34].

Finally, all the foregoing phenomena —spillover, competition between susceptible and resistant strains, and co-selection—are likely highly contextual, depending on patterns of between-host transmission, heterogeneous patterns of background antibiotic use [35], and the prevalence and relative fitness of the susceptible and resistant strains circulating in and around the treated community [14]. There is no guarantee that conclusions drawn from data collected in one context will be applicable in another context, especially when what precisely defines a distinct “context” remains unresolved.

### Statistical models of antibiotic use and resistance

**Fig. 1:**
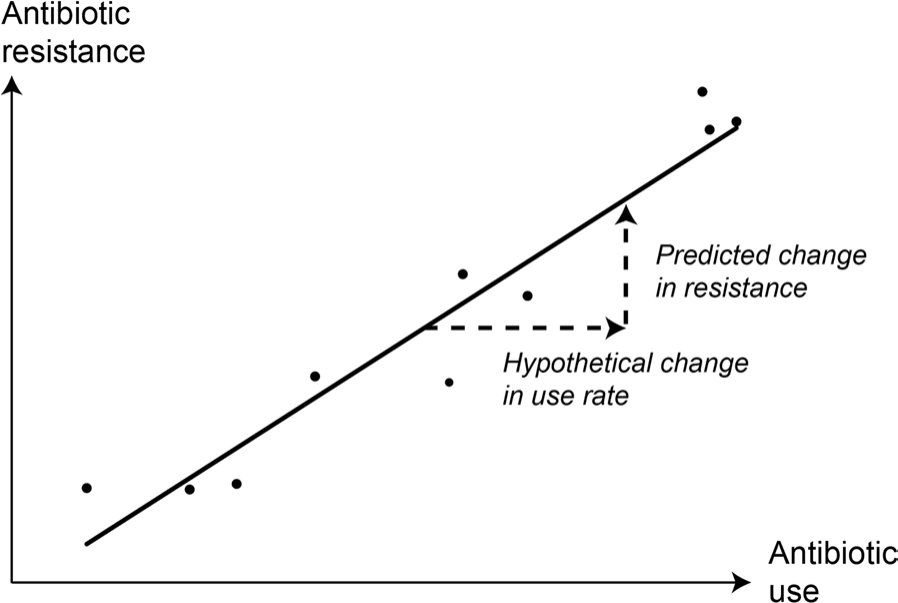
Conceptual schematic of a simple statistical model of antibiotic use and resistance. Empirical rates of population-level antibiotic use and resistance (points) are used to train a linear regression (line). Given a hypothetical increase in antibiotic use, the fit line can be used to predict the resulting change in population-level resistance

The saying goes: all models are wrong, but some are useful. A clearly “wrong” but parsimonious and potentially useful approach to modeling the complex relationship between antibiotic use and resistance is to infer the likely effects of a change in antibiotic use, such as MDA-azithromycin, using cross-sectional patterns of population-level antibiotic use and resistance (Fig. 1). In other words, in the absence of a complete understanding of the precise dynamics that relate population-level antibiotic use and resistance, one approach is to assume that the use-resistance associations observed in other contexts already incorporate these complexities [36] and then use those quantitative associations to predict the effects of MDA.

To illustrate this approach, compare MDA-azithromycin with outpatient azithromycin use in the US. Azithromycin use among American children aged 0‒2 years amounts to approximately 1 dose per year.^1^ Total US population-wide azithromycin use is approximately 700 doses per 1000 population per year.^2^ Thus, if MDA-azithromycin were instituted in the US on top of existing antibiotic use, then rates of azithromycin use among American children aged 0‒1 would triple, but total US azithromycin use would increase by less than 10%.^3^ For comparison, rates of population-wide macrolide use vary twofold across US states [23] and more than tenfold across European countries [37]. Thus, as a first approximation, the differences in macrolide resistance rates among US states and European countries might serve as an upper bound for the increase in population-level resistance that could be caused by MDA-azithromycin.

A conceptually similar but more quantitatively rigorous approach would be to fit a linear or quasibinomial regression to cross-sectional data about antibiotic use and resistance across US states or European countries. The regression model could then be used to estimate how a change in the input antibiotic use rate would affect the output antibiotic resistance rate [38] (Fig. 1). Statistical models need not be simple. However, generally speaking, given an input data set of measured rates of antibiotic use and resistance, and given an assumed mathematical relationship between use and resistance, a statistical modeling approach can find the parameters that best describe that mathematical relationship for that data set [39].

Statistical modeling has at least three fundamental limitations. First, it assumes that the processes that relate inter-country or inter-state differences in antibiotic use to differences in resistance are the same processes that govern how a perturbation in antibiotic use, such as MDA-azithromycin, would affect antibiotic rates [40]. In other words, the observed ecological use-resistance associations are assumed to be causal.

Second, statistical models can only attribute differences in resistance to differences in use or to other population-level covariates such as socioeconomic factors [38]. Statistical models are not designed to evaluate complex, mechanistic counterfactuals, such as whether different contact patterns could lead to different rates of antibiotic resistance in different subpopulations. Statistical models also cannot account for biological factors, unrelated to antibiotic use, that could drive changes in the prevalence of resistance [40]. The effects of these biological factors can manifest as secular trends in disease activity or resistance prevalence that appear unrelated with secular trends in antibiotic use. For example, secular trends in the prevalence of trachoma have been suggested as explanations for differences in the effect of MDA on *C. trachomatis* carriage across clinical trials [8, 29]. At the level of cities or countries, secular trends in antibiotic resistance can be on the order of 5 percentage points of collected isolates per year [41–43]. Smaller communities, like those targeted by MDA, might display different, and perhaps more rapid, dynamics that would likely not be accounted for in a straightforward statistical model.

Finally, a statistical model can only be built for antibiotics and pathogens for which there are pre-existing data. While the effects of azithromycin use on macrolide resistance among *S. pneumoniae* and *E. coli* are fairly well studied [1, 18, 27, 28], the effect of azithromycin use on resistance in other pathogens is poorly documented [44]. Comparing use-resistance associations across pathogens and antibiotics [21, 23, 45] may help fill in some gaps, but this approach is now only speculative.

The illustration above, comparing MDA-azithromycin with US azithromycin use, has many other weaknesses that could likely be ameliorated with more sophisticated statistical models. As one example, the crude model above only considers a single pathogen and antibiotic. A more careful approach would account for, or at least evaluate, the effect of the use of multiple antibiotics [34, 46, 47]. As a second example, the crude model does not account for the proportion of the population that is already receiving MDA-azithromycin as treatment for other pathogens like trachoma and so overestimates the increase in antibiotic use that would result from implementation MDA-azithromycin to reduce all-cause mortality.

### Mechanistic models of antibiotic use and resistance

Statistical models are likely useful for roughly estimating the absolute quantitative effect that MDA-azithromycin would have on population-level antibiotic resistance, but they cannot evaluate mechanistic questions or counterfactuals. Mechanistic models, on the other hand, make assumptions about the underlying dynamics that relate use and resistance. For example, a model might assume that a host can be colonized by only one strain of a bacterial species, either susceptible or resistant, while another model might assume that a host can be colonized by multiple strains at the same time. In either case, the model must specify factors like how the host immune system or antibiotic treatment will affect the colonizing bacteria.

**Fig. 2:**
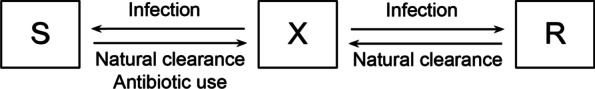
Simple mechanistic model of antibiotic use and resistance in a single population. Uncolonized individuals (*X*) can become colonized by the sensitive bacterial strain (*S*) or by the resistant strain (*R*). Sensitive- and resistant-colonized individuals can naturally clear colonization, for example, via host immunity. Antibiotic use leads to more rapid clearance among susceptible-colonized individuals. Compare Fig. 3A from Lipsitch et al. [50]

The most familiar mechanistic model of infectious disease is the classic susceptible-infected-recovered (SIR) model. A simple mechanistic model of antibiotic use and resistance is a susceptible-infected model, with two different infected compartments, one representing infection with the susceptible bacterial strain and the other representing the resistance strain (Fig. 2). Conceptually related but more complex mechanistic models have been used for decades to explore the link between antibiotic use and resistance [48, 49]. Through time, these models have developed greater theoretical soundness [25, 50] and greater complexity, including metapopulations representing geographic populations [51], age groups [52], and non-human environmental and animal compartments [53].

Mechanistic models could be adapted to evaluate the effect of antibiotic use in one population on resistance in another population [9] and then used for multiple study purposes. First, they could aid interpretation of MDA clinical trial data. For example, mechanistic models could identify factors that quantitatively explain the apparently disparate results in the MORDOR I study, in which MDA-azithromycin appeared to be more effective in reducing mortality in the Niger study population, compared to the populations in Malawi and Tanzania [3]. Second, mechanistic models could aid future experimental design by assessing what trial designs and sample sizes [54] would most efficiently gather information about the effect of MDA on resistance. Third, mechanistic models could be used to estimate the effects of different MDA implementations, such as comparing blanket treatment of all children in an age group versus targeting smaller “core” groups [17], or to explore the effect of repeated treatments on efficacy and resistance [55]. Finally, models could help estimate how other interventions, such as improvements in water, sanitation, and hygiene, would modulate MDA’s effects on antibiotic resistance.

**Fig. 3:**
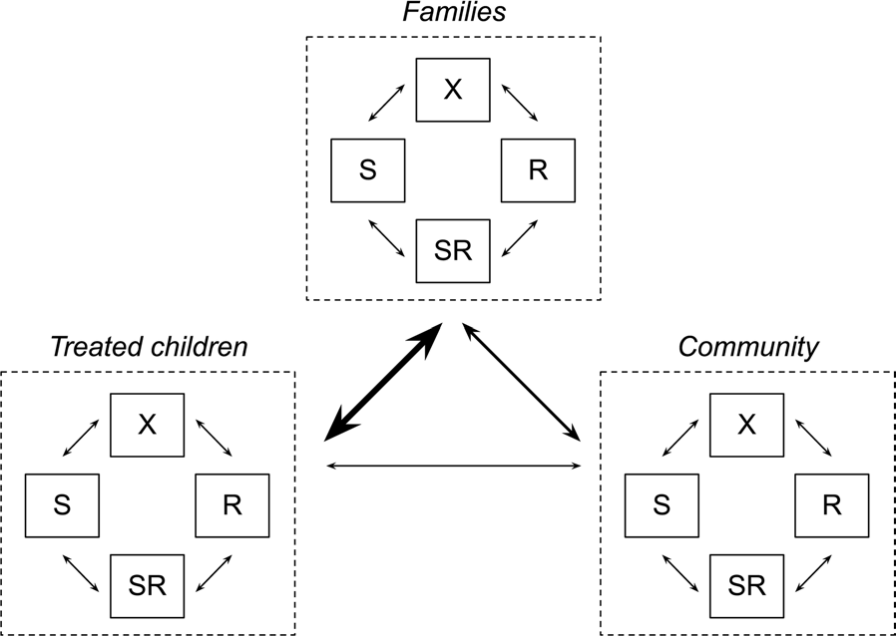
Conceptual schematic for a mechanistic model of mass drug administration. There are three host classes, representing the mass drug administration-treated children, their families, and the broader community. Members of each host class move between four colonization states: uncolonized (*X*), colonized by the sensitive bacterial strain (*S*), colonized by the resistant strain (*R*) or co-colonized (*SR*). Colonization dynamics in each host class can affect dynamics in other classes: children frequently exchange bacteria with their families (thick arrow) but less often with the broader community (thin arrow)

Mechanistic models of MDA-azithromycin would likely include three classes of hosts: first, the children treated with MDA; second, those children’s close contacts, such as family members and untreated children; and third, one or more further removed populations, such as other members of a settlement or the population of a larger administrative region (Fig. 3). Depending on the specificity required from the model, it may be important to further subdivide these compartments to account for differing patterns of transmission and immunity [52]. When modeling resistance among bacteria with environmental transmission routes, such as *E. coli*, it may be important to model environmental compartments, such as water sources [53]. While a greater number of host classes and environmental compartments allows for a more fine-grained assessment of the effects of resistance, more complex models are more difficult to parameterize and more likely to be mis-specified. Greater precision does not necessarily entail greater accuracy.

Contemporary models differ in their representation of the modeled bacteria (Fig. 3). Some models include just two strains of the same species, one susceptible and one resistant [25]; others track multiple sensitive and resistant strains, corresponding, for example, to *S. pneumoniae* serotypes [52, 56, 57]. In some models, a host can be colonized by only one strain, although there is increasing recognition of the importance of within-host competition between sensitive and resistant strains [14, 25]. More sophisticated bacterial dynamics, such as horizontal gene transfer, are beyond the scope of most contemporary modeling [14].

Mechanistic models of disease transmission are typically agent-based [52, 58] or compartmental [25, 53, 57, 59]. Agent-based models track individual people and the interactions between them, simulating bacterial transmission and changes in host colonization status. Compartmental models, on the other hand, track only each combination of host class and bacterial strain and assume that the individuals in each host class behave identically. While agent-based models allow for arbitrarily complex interaction networks and can straightforwardly simulate stochastic disease transmission trajectories, compartmental models are usually deterministic and more analytically tractable. For the purposes of modeling spillover resulting from MDA, compartmental modeling may be sufficient, for two reasons. First, the relevant transmission networks to be modeled might not be known to sufficient detail to merit the complexity of an agent-based approach. Second, uncertainty in model results may be due more to uncertainty in the input parameters rather than stochasticity in transmission chains. Thus, sensitivity analyses using deterministic models may be sufficient to faithfully characterize the possible range of model results, obviating the need to model stochastic disease trajectories with agent-based models.

Every process in a mechanistic model must be accompanied by a quantitative parameter, and models of antibiotic use and resistance for MDA will have many classes of parameters (Table 1). In many cases, the selection of parameter values can be informed by empirical data. Contact rates between different populations have been estimated in industrialized countries using surveys, commuting flows, and contact tracing [60–62], which can provide at least a rough estimate of the same patterns in settings where MDA may be implemented. Antibiotic use rates and vital dynamics could be estimated using local surveys [3]. Initial conditions could be informed by pre-MDA measurements of the prevalence of resistance in targeted communities. Bacterial clearance rates have been estimated for certain bacteria, especially *S. pneumoniae* [63–65]. In practice, however, these data are not sufficiently precise to confidently fix model parameters. Instead, models are typically fit to pre-existing antibiotic use and resistance data using Bayesian methods like Markov chain Monte Carlo [25, 57].

**Table 1:** Parameters likely required for mechanistic modeling of mass drug administration using bacterial transmission mechanics.

Parameter class	Number of parameters	Notes
Transmission rates (*β*)	*N* within-class and *N*-choose-2 between-class, where *N* is the number of host classes	Values depend on both host contact rates and probabilities of bacterial transmission per contact
Antibiotic use rates (*τ*)	1 per antibiotic and host class	More parameters are required if antibiotic use is explicitly time varying
Clearance rates (*u*)	1 per bacterial strain	Background processes of immunity or competition are assumed to clear bacteria from hosts
Resistance costs (*c*)	1 or 2 per resistant bacterial strain	Resistant strains are assumed to have lower transmission rates or higher clearance rates, relative to susceptible strains
Co-colonization parameters	Varies depending on co-colonization mechanisms	E.g., the model in Davies et al. [25] requires a co-colonization efficiency (*k*)
Initial conditions	1 per bacterial strain and host class	Starting prevalence of each strain
Vital dynamics	Varies depending on demographic model	Birth rates, migration rates, etc

The identity of these parameters and their notation was drawn from recent mechanistic models of use and resistance [25, 52, 58﻿]

To help quantify population-level effects of MDA, future MDA studies would measure rates of pathogen carriage and resistance to relevant antibiotics among individuals who are in the treated community but who are not treated themselves [5]. Ideally, these studies would also collect genotypic and phenotypic information on pathogen isolates, such as full antibiotic susceptibility profiles [66], multilocus sequence typing, or even whole genome sequences. In combination with linked host metadata, such as treatment status, age, family relationships, and location of residence, these pathogen data would further aid modeling of pathogen carriage and transmission specifically in settings where MDA is relevant [67, 68]. In practice, this kind of data collection is not feasible for every study, even in well-resourced settings. Modelers and clinical trialists should collaborate to identify the most resource-efficient approaches for collecting data that can address the most critical knowledge gaps about the effect of MDA on resistance.

Mechanistic modeling has important limitations. Population-level dynamics of resistance are complex, and models of resistance are not reliably predictive [14]. For MDA, the number of model parameters is likely large relative to the number of independent sampling units (i.e., MDA-treated populations) with data available for study. Without assurance that the model structure accurately reflects the underlying dynamics of bacterial transmission and competition, or that the parameter values are faithful to the setting to be modeled, mechanistic models’ quantitative predictions should be regarded with healthy skepticism. Instead, mechanistic models should be used as conceptual tools to “help us systematically examine the implications of various assumptions about a highly nonlinear process that is hard to predict using only intuition” [69].

### Limitations

It is worth noting that even in well-resourced settings like the US or Europe, the costs and benefits of antibiotic use have not been rigorously quantified. In general, antibiotic use is considered inappropriate when less intensive antibiotic therapy —a lower dose of antibiotics, a shorter regimen, a more narrow-spectrum antibiotic, an antibiotic less likely to select for problematic antibiotic resistance, or even no antibiotic at all— is expected to have the same clinical benefit [70–72]. If an individual patient will benefit from more antibiotics or stronger antibiotics, then those antibiotics’ effects on population-level antibiotic resistance are considered acceptable. This fact does not mean that a rigorous cost-benefit framework should not guide policy decisions about MDA-azithromycin, nor does it mean that we should not leverage all available data and methodologies, including modeling, to best estimate MDA’s benefits and costs. It only means that this rigorous evaluation will be a challenging and novel endeavor.

## Conclusions

There are many unknowns about the degree to which MDA selects for resistance, in whom, and at what cost. Future clinical studies can address some of these knowledge gaps. However, MDA studies cannot feasibly address the risk of resistance for every subgroup of patients [11, 12]. Mathematical modeling can help fill gaps in our knowledge using well-founded assumptions, especially if models are developed in coordination with decision-makers [73] and guided by well-formed experimental design options or authentic policy questions.

## Data Availability

Data sharing is not applicable to this article as no datasets were generated or analyzed during the current study.

## Abbreviation

MDA: Mass drug administration

## Footnotes

1 In 2011, children under 20 years old received 183 azithromycin prescriptions per 1000 population (75). Children under 2 received 48% more prescriptions than the average for all children aged 0‒20, and a typical course of azithromycin is 3 or 5 days (23), yielding 813 or 1354 annual doses per 1000 children aged 0‒2.

2 In 2011, Americans received 174 azithromycin prescriptions per 1000 population (75). Assuming 3 or 5 doses per prescription, this is 522 or 870 doses per 1000 population per year.

3 If 4% of the population is under 1 year and MDA covers 80% of those children, then 2 doses per child per year amounts to 64 doses per 1000 overall population per year, compared to the baseline of 700 doses per 1000 population per year.
